# Supplementary material for: Chromothripsis during telomere crisis is independent of NHEJ, and consistent with a replicative origin
Source: Genome Res. 2019 May;29(5):737–49. doi: 10.1101/gr.240705.118 (PMC6499312; doi:10.1101/gr.240705.118)
Supplement: Supplemental Material [file supp_gr.240705.118_Supplemental_file_1.zip › contigs/annotated_contigs/DB79/contig.2.DB79_length_590_mean_cov_8.76949152542.docx]

**DB79_length_590_mean_cov_8.76949152542**

```
TACTG|AAGTGCTCTTCAGTCAGCTGTGGTGAATGCTGCCAACCCCAGAACTCACCCTTCAGGGCAGTGGGCTCCCCTCTGGCCCACGA
      >chr1:180295496-180295615 + E=7e-60
CAAGTCCAGAAGGCTATCCAAGGCTTGTCTGGTCCA|TA|ATTGGGTTTCATAATTGGTTTCCATGATTCCATAACATACTACCAAACA
                                       >chr1:180197233-180197697 + E=1e-261
ATTTAATCTACTTCCCTAAGCCACTTTTCTTTTGTTGGCTATTTAGGTTATTTTTCTCCAAAACATAATACTGTAATAAACATTTTCAC

GCATGAAACTTTTATAAAAAATTCTAGATTATTTCCGTGGAATAGACATTCAAAAGTACTACATAAACTAAAGGGTATGAAAAATTTAT

GAGTTTGATACATATTGCCAAATTTCTTTCCAAACGGATGTTAAGAGTTTATAAGCACCCAGCAGTACTGGTGAGTGTCCAAGCCCCTT

TCGCCAGTCCTGAACTCTAGGATGCCCCTCCCGCGCGTCGGGCATCTCACAAACAGAAAAAGCTGTGCACGTGTTGTTTTGTCTTGCGT

GTTTTTCACTGCAAATGAGGCTGGCGTTTCCCCGTGTTTGCTTACTAGTTATATTTCTA
```
